# A new hand-held optical reflectometer to measure enamel erosion: correlation with surface hardness and calcium release

**DOI:** 10.1038/srep25259

**Published:** 2016-04-28

**Authors:** Thiago Saads Carvalho, Tommy Baumann, Adrian Lussi

**Affiliations:** 1 Department of Preventive, Restorative and Pediatric Dentistry, University of Bern, Freiburgstrasse 7, CH-3010, Bern, Switzerland

## Abstract

In the present study, the surface reflection intensity (SRI) was measured from enamel with different induced erosion degrees using a hand-held pen-size reflectometer (Hand-Held) and a Table-Top reflectometer. To validate the Hand-Held reflectometer, we correlated its optical signals with the change of surface microhardness (SMH), and amount of calcium released from the enamel samples during erosion. We used 124 tooth enamel specimens that were exposed to an erosive challenge of either 1, 2, 4, 6, 8, or 10 minutes. SRI and SMH were measured before and after the erosive challenge and we also measured the amount of calcium released to the citric acid. Relative SRI loss (rSRI_loss_) and relative SMH loss (rSMH_loss_) were calculated. rSRI_loss_ from the Hand-Held and the Table-Top reflectometers were similar and significantly correlated to rSMH_loss_ and calcium release. The regression analyses showed a significant association between rSRI_loss_ from both reflectometers and rSMH_loss_ and calcium, showing that both reflectometers can be used to measure erosive demineralization of enamel. The Hand-Held reflectometer is capable of assessing *in vitro* erosion, correlating to other commonly used methods. It is small, easy to handle and provides fast measurement, being a possible candidate to measure erosion in clinical studies.

By definition, erosive tooth wear is the chemical-mechanical process resulting in a cumulative loss of hard dental tissue not caused by bacteria^1^. More specifically, during an acidic challenge, hydrogen ions react with the tooth mineral leading to demineralization of the outermost surface of the enamel^2^. The enamel surface loses calcium ions^3^,^4^, loses hardness (surface softening)^5^,^6^, and becomes rougher, which leads to a decrease in surface reflection^7^.

It has long been accepted that initial enamel erosion can be accurately measured by surface microhardness measurements (SMH), since it is a robust method with a substantial number of studies published on the subject^8^. SMH, however, is not only a time-consuming method, but it also may not be sensitive enough to measure the very initial changes of the enamel surface during short-term erosive challenges. It is also semi-destructive method as it leaves indentations on the surface of the test sample and can be currently applied only *in vitro*. Another method used in erosion studies is atomic absorption spectroscopy (AAS). This method can be used to analyse the amount of calcium ions released from the tooth during an erosive challenge. However, while it is a sensitive method to quantify the amount of calcium released from the enamel surface, it provides no information on the morphological changes to the enamel surface. Additionally, it often requires normalizing the obtained calcium release per surface area of the enamel, which might be a challenge for the native curved dental surfaces. Because saliva also contains calcium ions, analysis of calcium release from enamel tissue only requires saliva-free experiments. This results in major applicability of AAS for *in vitro* studies, but limited utilization for *in vivo* erosion analysis.

Yet another method of quantifying dental erosion is the measurement of specular reflection from the enamel surface^7^. It is based on the fact that, when superficial and near-surface demineralization occurs, the loss of minerals from the tooth will lead not only to a softening, but also to a roughening of the surface. In a previous validation study, Rakhmatullina *et al.*^7^ used a reflectometer with a halogen light source coupled into a 600 μm fibre, which was able to record reflection signals in the range of 400–800 nm, at angles of incidence varying from 0° to 45°. The authors showed that specular reflection decreases as erosion progresses. The authors also showed that the specular reflection values measured with this first reflectometer correlated both to SMH and AAS analysis of calcium release^7^. In addition, this method can be used in comparative studies^9^, but the acquired enamel pellicle can influence the results^10^. More recently, the principle of this reflectometer was transferred and adjusted into a portable optical device^11^. This new hand-held reflectometer (Hand-Held) was developed for the purpose of using in the clinical setting and on native enamel surfaces with promising results^11^. Different to the reflectometer used by Rakhmatullina *et al.*^7^, the miniaturized Hand-Held version has a laser diode source of 635 nm, and the laser beam is coupled into a 105 μm fibre, and the angle of incidence is fixed at ~23°. Also recently, further changes were made to the original reflectometer, which now also consists of a laser diode (635 nm) with an incidence angle of 45°. This new reflectometer is herein named Table-Top device. Given the distinct differences in light sources and laser incidence angles between the reflectometer devices used by Rakhmatullina *et al.*^7^ and those used in the present manuscript (Table-Top and Hand-Held devices), it is indispensible that both devices are validated, not only against each other, but also against the SMH and AAS. Therefore, in the present study, the surface reflection intensity (SRI) was measured from enamel with different induced erosion degrees using the Hand-Held and the Table-Top reflectometers and these optical signals were correlated with the change of surface microhardness (SMH), and the amount of calcium released from the enamel samples during erosion.

## Results

**Figure 1:**
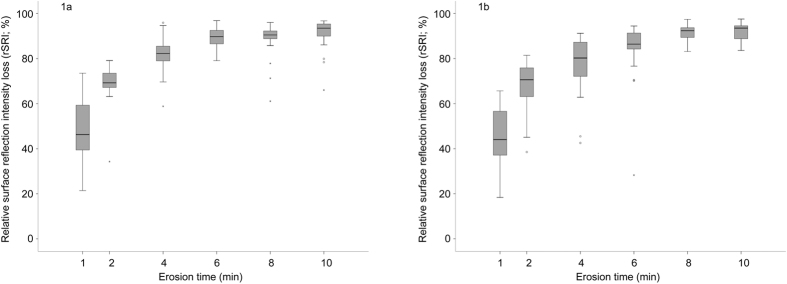
Relative surface reflection intensity loss (rSRI) from the Table-Top (1a) and Hand-Held (1b) devices after different erosion times.

As erosion time increased, we observed an increase in rSRI_loss_, rSMH_loss_ and calcium release. We used Spearman’s correlation coefficients (r_s_) to verify associations between the different methods (Table 1). The rSRI_loss_ results obtained using the Table-Top reflectometer (Fig. 1a) were significantly correlated (r_s_ = 0.81; p < 0.001) to those obtained with the Hand-Held reflectometer (Fig. 1b). The results showed an rSRI_loss_ of over 40% after only 1 minute of erosion, but rSRI_loss_ steadily increased up to 6 minutes of erosion, when both reflectometers registered an rSRI_loss_ of above 80%. Thereafter, no further noticeable change in rSRI_loss_ was observed with either device (Fig. 1a,b).

**Figure 2:**
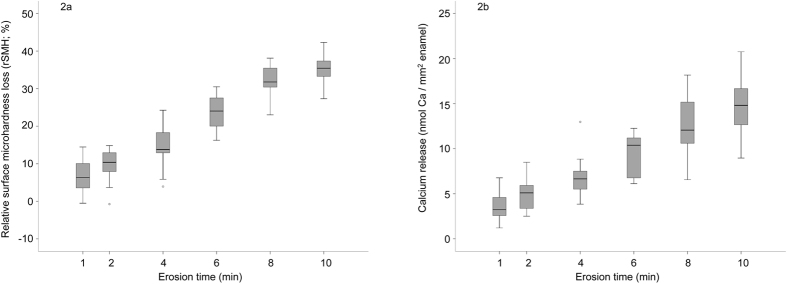
Relative surface microhardness loss (rSMH; 2a) and calcium released from the specimens (2b) after different erosion times.

rSMH_loss_ (Fig. 2a) also increased with erosion time. Within the first 2 minutes of erosion, the samples lost approximately 10% of their original hardness, whereas after 8 minutes of erosion the specimens presented more than a 30% loss of the original hardness (Fig. 2a). Similarly to rSMH_loss_, calcium release also increased as erosion time increased (Fig. 2b).

**Figure 3:**
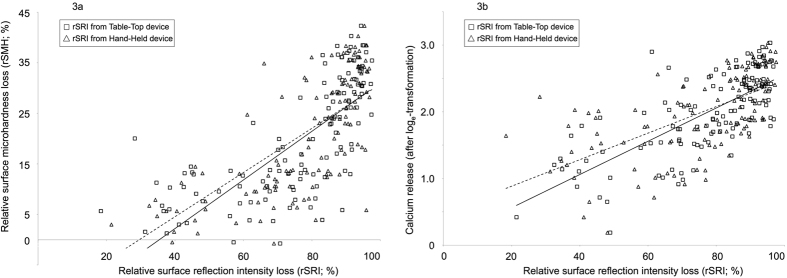
Association between the relative surface reflection intensity loss (rSRI) from the Table-Top (◻) and Hand-Held (△) devices with relative surface microhardness loss (3a) and calcium (after log_e_-transformation) released from the specimens (3b). Solid line: regression for Table-Top device; dashed line: regression for Hand-Held device.

Figure 3 presents the association between rSRI_loss_ from the Table-Top (squares) and Hand-Held (triangles) reflectometers with the rSMH_loss_ values (Fig. 3a) and calcium release values (Fig. 3b). We observed a significant correlation (p < 0.001) between rSRI_loss_ and rSMH_loss_ results (r_s_ = 0.77 and r_s_ = 0.79 for the Table-Top and Hand-Held reflectometers, respectively; Fig. 3a). Furthermore, the linear regression analyses (Fig. 3a) showed a significant association between rSRI_loss_ data from both reflectometers and rSMH_loss_ values, with regression equations (1) and (2) fitting the data:


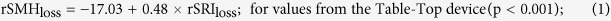



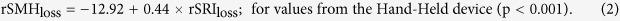


Similarly, a significant correlation (p < 0.001) was observed between calcium release and rSRI_loss_ data from both reflectometers (r_s_ = 0.69 and r_s_ = 0.70 for the Table-Top and Hand-Held reflectometers, respectively). Also, after log_e_-transformation of the calcium results (ln[Ca]), linear regression analyses (Fig. 3b) showed a significant association between rSRI_loss_ data from both reflectometers and calcium values, with regression equations (3) and (4) fitting the data:


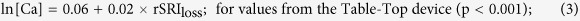



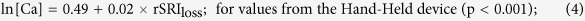


## Discussion

In keeping with the results of previous studies^7^,^9^,^10^,^11^, which focussed on the use of the Table-Top reflectometer, the present results also showed that SRI (measured with the Hand-Held reflectometer) was well correlated with other methods commonly used to measure enamel erosion *in vitro*: SMH and calcium release. During an erosive challenge, the surface and near-surface enamel is partially demineralized, causing the release of calcium ions to the erosive solution, as well as a decrease in surface hardness^3^,^8^,^12^ and an increase in surface roughness^13^. Consequently, after an erosive challenge, the enamel surface has a typical honeycomb pattern, as reported by Meurman and Frank^14^. Also, a rougher surface presents lower SRI values^7^,^9^,^10^,^11^.

Rakhmatullina *et al.*^7^ and Lussi *et al.*^6^ discussed that erosion occurs in two stages. During the first stage of erosion, the acid causes a softening of the near-surface layer of enamel (softening stage), which occurs within the first few minutes of incubation in the acid. As the acid impact continues with longer erosion times, the demineralization eventually leads to loss of the enamel surface (surface loss stage). Both stages cause a substantial increase in the enamel surface roughness and considerable decrease in surface reflectivity. Similarly to what was reported by Rakhmatullina *et al.*^7^, we have observed that the initial surface softening stage occurred within the first 6 minutes of erosion. After 6 minutes, enamel surface loss was already observed (results not shown). This coincides with a decrease in sensitivity of the Hand-Held and Table-Top reflectometers, which is clearly observed in Figures 1a and 1b, where the rSRI_loss_ increases up to 6 minutes of erosion, but tends to level out afterwards. Therefore, we show that both reflectometers were markedly more sensitive during the initial stages of enamel erosion, i.e. only during the enamel softening stage.

A satisfactory correlation between SRI (measured with the Table-Top reflectometer) and other parameters has already been reported by Rakhmatullina *et al.*^7^ and Brevik *et al.*^9^, who observed high correlation values of r^2^ = 0.70–0.92 between SRI and SMH, and r^2^ = 0.68–0.93 between SRI and calcium release. The present study also reports the high correlation coefficient values between SRI from the new Hand-Held reflectometer and SMH or calcium release (in the range of ~0.7 to ~0.8). These satisfactory values render the device satisfactory for future use in dentistry. In addition to the high correlation values, the formulas for the regression analyses show the close relationship between rSMH_loss_ or calcium release and the rSRI_loss_ results. Previous studies have already shown that the Table-Top device can be used to measure erosive demineralization in the laboratory. However, results obtained with the Hand-Held reflectometer strongly indicate that it might provide measurements of dental erosion already on the very early stage. This reflectometer is a compact pen-size device, but the angle of incidence had to be reduced to 23° instead of the 45° from the Table-Top device. Initial measurements, however, showed comparable results between the two^11^. Also, the miniaturized reflectometer does not require significant space in the laboratory table compared to Table-Top set-up, and the easy-handling of the device can produce fast measurements of the tooth’s surface. Furthermore, there is currently no device available to aid clinicians in the diagnosis of initial dental erosion in clinical settings. Since the Hand-Held reflectometer showed a significantly good correlation with the other methods, this device can be seen as a good possibility to measure the initial stages of erosion in clinical research, especially in *in situ* experiments.

Clinically, erosive tooth wear is the combination of erosion and abrasion, and all teeth are constantly exposed to acid and mechanical impacts. So, in clinical settings, mechanical impacts, such as tooth brushing^15^ or abrasion from the tongue^16^ or from hard food sources, will partly remove the rougher and softer (demineralized) enamel^17^, leaving a smoother aspect to the tooth surface with higher SRI values^10^. In this case, eroded teeth have a shiny, silky-glazed, but sometimes dull appearance, with excessively smooth surfaces due to the absence of perikymata^6^,^18^. Hence, we suggest that SRI values will be clinically higher for eroded teeth, because of their highly polished surface, and lower for sound teeth, because of the presence of perikymata that contribute to a rougher aspect of the surface. Although further tests on native enamel surfaces are necessary, our results suggest that the Hand-Held reflectometer can be used to measure erosion in the laboratory and *in situ*, and might also be a useful tool in the future, to aid clinicians in diagnosing the early stages of erosive tooth wear. So, we conclude that the Table-Top and Hand-Held reflectometers presented similar rSRI_loss_ measurements, and a high correlation with rSMH_loss_ and calcium release.

## Materials and Methods

### Enamel sample preparation

From a pool of extracted teeth, a total of 124 caries-free human premolars were selected. The teeth were extracted by dental practitioners in Switzerland and they were kept in a 2% Chloramine solution until the time of the experiment. Before the extraction, the patients were informed about the use of their teeth for research purposes and their oral consent was obtained. Because we are using teeth from a pooled bio-bank, the local ethics committee categorized the samples as “irreversibly anonymised”, and no previous approval was necessary. The present experiment was carried out in accordance with the approved guidelines and regulations of the local ethical committee (Kantonale Ethikkommission: KEK).

The enamel specimens were coated with a layer of coloured nail varnish for later determination of the exposed enamel area. The specimens were embedded in resin (Paladur, Heraeus Kulzer GmbH, Hanau, Germany) using two planar parallel moulds. The thinner mould (200 μm thick) determined how much enamel was ground away during the polishing procedure. For polishing, the enamel specimens were serially abraded under constant tap water cooling using a Knuth Rotor machine (LabPol 21, Struers, Copenhagen, Denmark) with silicon carbide paper discs of grain size 18.3 μm, 8 μm, and 5 μm for 60 seconds each. Further polishing for 60 seconds with 3 μm diamond abrasive was carried out with Struers polishing cloth under constant cooling (LaboPol-6, DP-Mol Polishing, DP-Stick HQ, Struers). Between each abrading and polishing step, as well as after the final polishing step, all enamel slabs were sonicated for 1 minute in water. This procedure removed exactly 200 μm of the surface enamel, leaving a flat and smooth surface area for the experiment. All samples were stored in a mineral solution (1.5 mmol/l CaCl_2_, 1.0 mmol/l KH_2_PO_4_, 50 mmol/l NaCl, pH = 7.0)^19^. Immediately prior to the experiment, the specimens were submitted to a final polishing step with 1 μm diamond abrasive paste (60 seconds, LaboPol-6, DP-Mol Polishing, DP-Stick HQ, Struers, Copenhagen, Denmark) and sonicated for 60 seconds, and randomly divided into six groups, according to the time of the erosive challenge.

### Experimental procedure and erosive challenge

Initially, we measured SRI and SMH of all specimens. The specimens were then submitted to one erosive challenge of either 1, 2, 4, 6, 8, or 10 minutes. The erosive challenge consisted of individually immersing the specimen in 10 ml of citric acid (1% citric acid, pH = 3.6, 25 °C, constant agitation of 70 rpm). After removing the specimens from the acid, we rinsed them in deionized water for 20 seconds and then dried with air (5 seconds). Lastly, we measured the amount of calcium released in the citric acid probes, and we measured the SRI and SMH again.

### Enamel surface reflection intensity (SRI)

We measured enamel SRI using two devices: one hand-held pen-size^11^ reflectometer (Hand-Held) and one Table-Top^7^ reflectometer. Each device was connected to its own computer running a specific software program used to register SRI. Initially, we measured SRI in all samples using the Table-Top reflectometer. For those measurements, the samples were individually placed on a platform (sample holder) under a laser beam, the source of which is fixed to the reflectometer. As previously described^7^, the samples are placed on the sample holder platform, and the laser beam is adjusted on the sample surface by moving the holder in the z-axis (height). The point of highest reflection intensity is then registered, which represents the best positioning of the sample in relation to the device. Later, we measured SRI using the Hand-Held reflectometer. This pen-sized device functions in a similar manner, but the laser beam derives from the hand-held piece, the tip of which is placed directly onto the enamel surface. In the same way that the sample in the Table-Top device moves along the z-axis, the laser from the Hand-Held reflectometer is also adjusted on the surface of the sample by slightly inclining the device in different angles, and the point of highest reflection intensity is then registered^11^. For both the Table-Top and Hand-Held reflectometers, the point of highest reflection intensity is expressed as an SRI value, which represents the best positioning of the sample in relation to the reflectometer. Highest SRI value corresponds to non-eroded polished enamel surface, and as the enamel surface is eroded, the SRI value decreases.

The reflectometers are each fitted with a laser diode (oeMarket, Cherrybrook, Australia), which emits a laser beam (635 nm) onto the surface of the sample. The reflected light is then captured and measured with a photodiode (FDS100, Thorlabs, Dachau, Germany). The Table-Top device has a beam incidence angle of 45°, whereas the Hand-Held device has an angle of incidence if ~23°.

For the statistical analyses in the present experiment, we calculated the relative SRI loss (rSRI_loss_) for each reflectometer. Since SRI measurements were carried out using both reflectometers at baseline (SRI_initial_) and after the erosive challenge (SRI_final_), we calculated the rSRI_loss_ for both devices using the formula: rSRI_loss_ = ((SRI_initial_ − SRI_final_)/SRI_initial_) × 100.

### Enamel surface microhardness (SMH)

SMH measurements were performed using a Knoop diamond under a load of 50 gf and a dwell time of 10 seconds (UHL VMHT Microhardness Tester, UHL Technischer Mikroskopie, Aßlar, Germany). For every hardness measurement, six indentations per specimen were made at intervals of 25 μm on a defect-free surface area. The length of the long axis of each indentation was measured and the hardness value automatically calculated. The mean hardness value from the 6 indentations was considered as the “true” hardness value for the specimen. SMH measurements were carried out at the beginning of the experiment (SMH_initial_), and after the erosive challenge (SMH_final_). For statistical analyses, we calculated the relative SMH loss (rSMH_loss_) after the erosive challenge using the formula rSMH_loss_ = ((SMH_initial_ - SMH_final_)/SMH_initial_) × 100.

### Analysis of calcium release

In order to quantify the amount of calcium released during the erosive challenge, the enamel surface area was measured. For these measurements, we used a light microscope (Leica, M420) with a fixed camera (Leica, DFC495). The contour between the exposed enamel area (white) and the colored nail varnish was traced, and the enamel surface area was measured (in mm^2^) using the software program IM500. This measurement was used to normalize the values of calcium released into the citric acid probes per enamel surface area.

We measured the calcium concentrations in the citric acid probes by means of a flame atomic absorption spectrometer (AAnalyst 400, Perkin Elmer Analytical Instruments, Shelton, USA). Standards of known calcium concentrations (Titrisol^®^, Merck, Darmstadt, Germany) were used to calibrate the instrument and obtain a working curve. Lanthanum nitrate was added to the citric acid and to the standards (final concentration 0.5% w/v) to eliminate interference from phosphate ions. The calcium concentration in each citric acid probe was measured and then normalized to the area of the enamel surface exposed to the acid. The calcium released is, therefore, expressed in nmol/mm^2^ enamel.

### Statistical analyses

Spearman’s correlation coefficients (r_s_) were calculated between the different methods used to measure erosion. We also performed linear regressions between rSRI_loss_ (from each of the two optical devices) and calcium release, as well as between rSRI_loss_ (from each of the two optical devices) and rSMH_loss_. Significance levels were considered at 0.05 and p-values are given up to 3 decimal places (p-values lower than 3 decimal places are simply given as p < 0.001).

## Additional Information

**How to cite this article**: Carvalho, T. S. *et al.* A new hand-held optical reflectometer to measure enamel erosion: correlation with surface hardness and calcium release. *Sci. Rep.* **6**, 25259; doi: 10.1038/srep25259 (2016).

## Figures and Tables

**Table 1:** Summary of the Spearman’s correlation coefficient values (r_s_) and their respective significances (p-values), for the association between the different methods used to measure erosion.

	rSRI_loss_ Table-Top	rSMH_loss_	Calcium Release
rSRI_loss_ Hand Held	0.81 p < 0.001	0.79 p < 0.001	0.70 p < 0.001
rSMH_loss_	0.77 p < 0.001	–	–
Calcium Release	0.69 p < 0.001	–	–
